# Supplementary material for: Conflation between knowledge and acceptance may contribute to the knowledge gap between Judeo-Christian and non-religious people
Source: PLoS One. 2026 Jan 2;21(1):e0319962. doi: 10.1371/journal.pone.0319962 (PMC12758684; doi:10.1371/journal.pone.0319962)
Supplement: S1 Table — All factor loadings are significant at p < .001. (DOCX) [file pone.0319962.s001.docx]

**S1 Table**. **Confirmatory Factor and Reliability Analysis for Survey Latent Variables**. All factor loadings are significant at p<.001.

| **Constructs** | | **Factor Loading** | |
| --- | --- | --- | --- |
| **Knowledge of Evolution** (EALS-SF; Short & Hawley, 2012)  Fit: RMSEA = 0.099, CFI = 0.989, TLI = 0.977, SRMR = 0.015  Scale: Strongly Agree, Agree, Somewhat Agree, Somewhat Disagree, Disagree, Strongly Disagree | | | |
| *In most populations, more offspring are born than can survive. (removed)* | | 0.360 | |
| *Mutations can be passed down to the next generation.* | | 0.808 | |
| *Increased genetic variability makes a population more resistant to extinction.* | | 0.760 | |
| *The more recently species share a common ancestor, the more closely related they are.* | | 0.753 | |
| *Mutations occur all the time.* | | 0.785 | |
| **Religiosity** (Sethi & Seligman, 1993; Manwaring et al., 2015; Jensen et al., 2019)  CFA is for religious individuals only.  Fit: RMSEA = .079, CFI=.962, TLI=.945, SRMR=.070  Correlated errors: Religious Practice Item 1 with Item 3; Religious Influence Items 1 with 2, and 3 with 4. | | | |
| Religious Practice  Scale: More than once a day, Once a day, More than once a week, Once a week, More than once a month, Less than once a month | | | |
| *How often do you read holy scriptures?* | | 0.730 | |
| *How often do you attend Sunday School, religious classes, or seminars?* | | 0.878 | |
| *How often do you pray?* | | 0.497 | |
| *How often do you attend organize worship services?* | | 0.870 | |
| *How often do you attend other activities sponsored by a religious group?* | | 0.710 | |
| Religious Influence  Scale: No influence at all, Hardly any influence, Some influence, Moderate influence, A lot of influence, Extreme influence | | | |
| *How much influence do your religious beliefs have on what you wear?* | | 0.773 | |
| *How much influence do your religious beliefs have on what you eat and drink?* | | 0.716 | |
| *How much influence do your religious beliefs have on your choices about whom you associate with?* | | 0.840 | |
| *How much influence do your religious beliefs have on what social activities you undertake?* | | 0.911 | |
| *To what extent do your religious beliefs impact the important decisions that you make?* | | .0828 | |
| (Religious Hope was omitted due to lack of fit) | | | |
| **Acceptance of Evolution** (I-SEA; Nadelson & Southerland, 2012)  Fit (Religious): RMSEA = .071, CFI=.918, TLI=.907, SRMR=.055  Correlated errors: Human Item 1 with Item 4; Human Item 4 with Item 7  Fit (Non-Religious): RMSEA = .061, CFI=.908, TLI=.895, SRMR=.047  Correlated errors: Macro Item 2 with Item 3; Micro Item 1 with Item 4; Human Item 2 with Items 3 and 5; Human Item 4 with Item 7  Scale: Strongly Agree, Agree, Somewhat Agree, Somewhat Disagree, Disagree, Strongly Disagree | | | |
| *Macroevolution* | Relig. | | Non. |
| *I think that new species evolved from ancestral species.* | 0.748 | | 0.787 |
| *I think that the fossil evidence that scientists use to support evolutionary theory is weak and inconclusive.* | -0.827 | | -0.741 |
| *There are a large number of fossils all around the world that support the idea that organisms evolve into new species over time.* | 0.905 | | 0.779 |
| *I think all complex organisms evolved from single celled organisms.* | 0.696 | | 0.676 |
| *I think that new species evolve from a lot of small changes occurring over relatively long periods of time.* | 0.848 | | 0.682 |
| *There is little or no observable evidence to support the theory that describes how one species of organism evolves from a different ancestral form.* | -0.779 | | -0.818 |
| *The forms and diversity of organisms have changed dramatically over time.* | 0.743 | | 0.672 |
| *I think that all organisms are related (or share a common ancestor)* | 0.547 | | n/a |
| *Microevolution* | | | |
| *I think that organisms, as they exist now, are perfectly adapted to their natural environments and so will not continue to change.* | 0.689 | | 0.526 |
| *All groups of organisms will continue to change.* | -0.799 | | -0.610 |
| *There are a large number of examples of organisms that have undergone evolutionary changes with the species (i.e., antibiotic resistance in bacteria, production of new strains of the flu virus).* | -0.792 | | -0.825 |
| *Species were created to be perfectly suited to their environment, so they do not change.* | 0.778 | | 0.653 |
| *I don't accept the idea that a species of organism will evolve new traits over time.* | 0.719 | | 0.711 |
| *I think there is an abundance of observable evidence to support the theory describing how variations within a species can happen.* | -0.757 | | -0.770 |
| *Species exist today in exactly the same shape and form in which they always have.* | 0.707 | | 0.527 |
| *There is overwhelming evidence supporting the theory of evolution to explain how variations in a species develop over time.* | -0.851 | | -0.839 |
| *Human Evolution* | | | |
| *There is reliable evidence to support the theory that describes how humans were derived from ancestral primates.* | 0.835 | | 0.852 |
| *Although humans may adapt, humans have not/do not evolve.* | -0.903 | | -0.674 |
| *I think the physical structures of humans are too complex to have evolved.* | -0.902 | | -0.744 |
| *I think that humans and apes share an ancient ancestor.* | 0.841 | | 0.863 |
| *I think that humans evolve.* | 0.923 | | 0.798 |
| *Humans do not evolve; they can only change their behavior.* | -0.903 | | -0.666 |
| *The many characteristics that humans share with other primates (i.e., chimpanzees, gorillas) can best be explained by our sharing a common ancestor.* | 0.791 | | 0.860 |
| *Physical variations in humans (i.e., eye color, skin color) were derived from the same processes that produce variation in other groups of organisms.* | n/a | | 0.765 |
