# Supplementary material for: Conflation between knowledge and acceptance may contribute to the knowledge gap between Judeo-Christian and non-religious people
Source: PLoS One. 2026 Jan 2;21(1):e0319962. doi: 10.1371/journal.pone.0319962 (PMC12758684; doi:10.1371/journal.pone.0319962)
Supplement: S1 File — (DOCX) [file pone.0319962.s002.docx]

**S1 File. Survey Instrument for Nationwide Survey**

**Filters:**

1. What is your age?
   1. Under 18
   2. 18 or older

[skip logic: if “Under 18”, skip to the end]

1. Which of the following best describes your current religious orientation?
   1. Agnostic
   2. Atheist
   3. Baptist
   4. Catholic
   5. Christian
   6. Church of Christ/Disciples of Christ
   7. Church of Jesus Christ of Latter-day Saints
   8. Congregational
   9. Episcopalian/Anglican
   10. Jehovah’s Witness
   11. Jewish
   12. Lutheran
   13. Methodist/Wesleyan
   14. Muslim
   15. Orthodox (Eastern)
   16. Pentecostal/Charismatic
   17. Protestant (Other)
   18. Reformed/Presbyterian
   19. Seventh-day Adventist
   20. Other Christian not listed above

**Demographics**

1. What is your education level?
   1. High school only
   2. Some college
   3. Associates degree
   4. 4-year college degree
   5. Graduate/Professional Degree
2. What is your gender?
   1. Male
   2. Female
   3. Other
3. What is your political ideology?
   1. Very Conservative
   2. Conservative
   3. Moderate Conservative
   4. Moderate
   5. Moderate Liberal
   6. Liberal
   7. Very Liberal

**Scientific Reasoning Ability**

1. Suppose you are given two clay balls of equal size and shape. The two clay balls also weigh the same. One ball is flattened into a pancake-shaped piece. *Which of these statements is correct*?
   1. The pancake-shaped piece weighs more than the ball
   2. The two pieces still weigh the same
   3. The ball weighs more than the pancake-shaped piece
2. This is because (referring to the question directly above)
   1. The flattened piece covers a larger area.
   2. The ball pushes down more on one spot.
   3. When something is flattened it loses weight.
   4. Clay has not been added or taken away.
   5. When something is flattened it gains weight.
3.
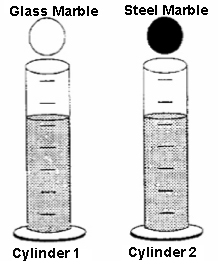
To the right are drawings of two cylinders filled to the same level with water. The cylinders are identical in size and shape. Also shown at the right are two marbles, one glass and one steel. The marbles are the same size but the steel one is much heavier than the glass one. When the glass marble is put into Cylinder 1 it sinks to the bottom and the water level rises to the 6^th^ mark. *If we put the steel marble into Cylinder 2, the water will rise*
   1. To the same level as it did in Cylinder 1.
   2. To a higher level than it did in Cylinder 1.
   3. To a lower level than it did in Cylinder 1.
4. This is because (referring to the question directly above)
   1. The steel marble will sink faster.
   2. The marbles are made of different materials.
   3. The steel marble is heavier than the glass marble.
   4. The glass marble creates less pressure.
   5. The marbles are the same size.
5.
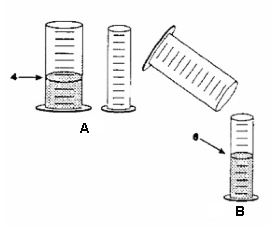
To the right are drawings of a wide and a narrow cylinder. The cylinders have equally spaced marks on them. Water is poured into the wide cylinder up to the 4^th^ mark (see A). This water rises to the 6^th^ mark when poured into the narrow cylinder (see B). Both cylinders are emptied (not shown) and water is poured into the wide cylinder up to the 6^th^ mark. *How high would this water rise if it were poured into the empty narrow cylinder*?
   1. To about 8
   2. To about 9
   3. To about 10
   4. To about 12
   5. None of these answers is correct
6. This is because (referring to the question directly above)
   1. The answer cannot be determined with the information given.
   2. It went up 2 more before, so it will go up 2 more again.
   3. It goes up 3 in the narrow for every 2 in the wide.
   4. The second cylinder is narrower.
   5. One must actually pour the water and observe to find out.
7. Water is now poured into the narrow cylinder (described in the question above) up to the 11^th^ mark. *How high would this water rise if it were poured into the empty wide cylinder*?
   1. To about 7 $1/2$
   2. To about 9
   3. To about 8
   4. To about 7 $1/3$
   5. None of these answers is correct
8. This is because (referring to the question directly above)
   1. The ratios must stay the same.
   2. One must actually pour the water and observe to find out.
   3. The answer cannot be determined with the information given.
   4. It was 2 less before so it will be 2 less again.
   5. You subtract 2 from the wide for every 3 from the narrow.
9. At the right are drawings of three strings hanging from a bar. The three strings have metal weights attached to their ends. String 1 and String 3 are the same length. String 2 is shorter. A 10-unit weight is attached to the end of Strings 1 and 2. A 5-unit weight is attached to the end of String 3. The strings (and attached weights) can be swung back and forth and the time it takes to make a swing can be timed. Suppose you want to find out whether the length of the string has an effect on the time it takes to swing back and forth. *Which strings would you use to find out*?
   1.
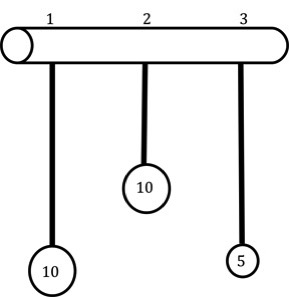
Only one string
   2. All three strings
   3. 2 and 3
   4. 1 and 3
   5. 1 and 2
10. This is because (referring to the question directly above)
    1. You must use the longest strings.
    2. You must compare strings with both light and heavy weights.
    3. Only the lengths differ.
    4. To make all possible comparisons.
    5. The weights differ.
11. Twenty fruit flies are placed in each of four glass tubes. The tubes are sealed. Tubes I and II are partially covered with black paper; Tubes III and IV are not covered. The tubes are placed as shown. Then they are exposed to red light for five minutes. The number of flies in the uncovered part of each tube is shown in the drawing. *This experiment shows that flies respond to* (response means move to or away from)


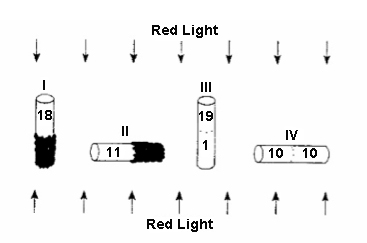


- 1. Red light but not gravity
  2. Gravity but not red light
  3. Both red light and gravity
  4. Neither red light nor gravity

1. This is because (referring to the question directly above)
   1. Most flies are in the upper end of Tube III but spread about evenly in Tube II.
   2. Most flies did not go to the bottom of Tubes I and III.
   3. The flies need light to see and must fly against gravity.
   4. The majority of flies are in the upper ends and in the lighted ends of the tubes.
   5. Some flies are in both ends of each tube.
2. In a second experiment, a different kind of fly and blue light was used. The results are shown in the drawing. *These data show that these flies respond to* (respond means move to or away from)


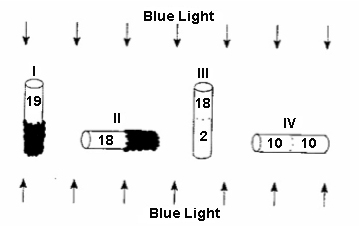


- 1. Blue light but not gravity
  2. Gravity but not blue light
  3. Both blue light and gravity
  4. Neither blue light nor gravity

1. This is because (referring to the question directly above)
   1. Some flies are in both ends of each tube.
   2. The flies need light to see and must fly against gravity.
   3. The flies are spread about evenly in Tube IV and in the upper end of Tube III.
   4. Most flies are in the lighted end of Tube II but do not go down in Tubes I and III.
   5. Most flies are in the upper end of Tube I and the lighted end of Tube II.
2.
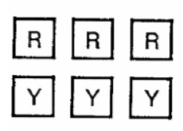
Six square pieces of wood are put into a cloth bag and mixed about. The six pieces are identical in size and shape, however, three pieces are red and three are yellow. Suppose someone reaches into the bag (without looking) and pulls out one piece. *What are the chances that the piece is red*?
   1. 1 chance out of 6
   2. 1 chance out of 3
   3. 1 chance out of 2
   4. 1 chance out of 1
   5. cannot be determined
3. This is because (referring to the question directly above)
   1. 3 out of 6 pieces are red.
   2. There is no way to tell which piece will be picked.
   3. Only 1 piece of the 6 in the bag is picked.
   4. All 6 pieces are identical in size and shape.
   5. Only 1 red piece can be picked out of the 3 red pieces.
4. Three red square pieces of wood, four yellow square pieces, and five blue square pieces are put into a cloth bag. Four red round pieces, two yellow round pieces, and three blue round pieces are also put into the bag. All the pieces are then mixed about. Suppose someone reaches into the bag (without looking and without feeling for a particular shape piece) and pulls out one piece. *What are the chances that the piece is a red round or blue round piece*?
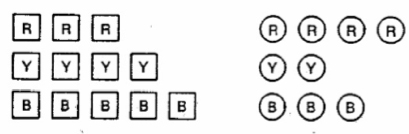

   1. Cannot be determined
   2. 1 chance out of 3
   3. 1 chance out of 21
   4. 15 chances out of 21
   5. 1 chance out of 2
5. This is because (referring to the question directly above)
   1. 1 of the 2 shapes is round.
   2. 15 of the 21 pieces are red or blue.
   3. There is no way to tell which piece will be picked.
   4. Only 1 of the 21 pieces is picked out of the bag.
   5. 1 of every 3 pieces is a red or blue round piece.
6. Farmer Brown was observing the mice that live in his field. He discovered that all of them were either fat or thin. Also, all of them had either black tails or white tails. This made him wonder if there might be a link between the size of the mice and the color of their tails. So he captured all of the mice in one part of his field and observed them. Below are the mice that he captured. *Do you think there is a link between the size of the mice and the color of their tails*?
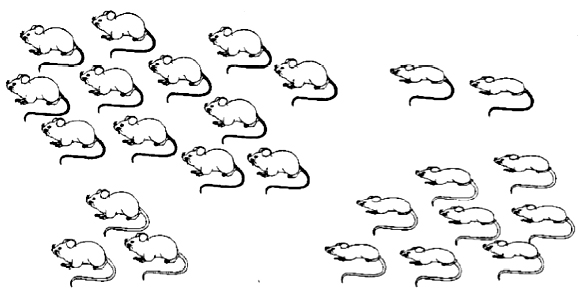

   1. Appears to be a link
   2. Appears not to be a link
   3. Cannot make a reasonable guess
7. This is because (referring to the question directly above)
   1. There are some of each kind of mouse.
   2. There may be a genetic link between mouse size and tail color.
   3. There were not enough mice captured.
   4. Most of the fat mice have black tails while most of the thin mice have white tails.
   5. As the mice grew fatter, their tails became darker.
8. The figure below at the left shows a drinking glass and a burning birthday candle stuck in a small piece of clay standing in a pan of water. When the glass is turned upside down, put over the candle, and placed in the water, the candle quickly goes out and water rushes up into the glass (as shown at the right). This observation raises an interesting question: Why does the water rush up into the glass?
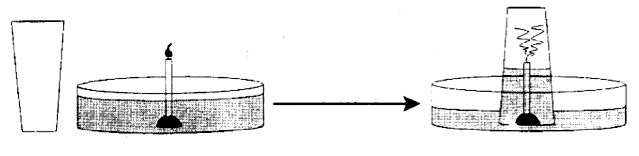


Here is a possible explanation. The flame converts oxygen into carbon dioxide. Because oxygen does not dissolve rapidly into water but carbon dioxide does, the newly formed carbon dioxide dissolves rapidly into the water, lowering the air pressure inside the glass.

Suppose you have the materials mentioned above plus some matches and some dry ice (dry ice is frozen carbon dioxide). *Using some or all of the materials, how could you test this possible explanation*?

1. Saturate the water with carbon dioxide and redo the experiment noting the amount of water rise.
2. The water rises because oxygen is consumed, so redo the experiment in exactly the same way to show water rise due to oxygen loss.
3. Conduct a controlled experiment varying only the number of candles to see if that makes a difference.
4. Suction is responsible for the water rise, so put a balloon over the top of an open-ended cylinder and place the cylinder over the burning candle.
5. Redo the experiment, but make sure it is controlled by holding all independent variables constant; then measure the amount of water rise.
6. What result of your test (mentioned above) would show that your explanation is probably wrong?
   1. The water rises the same as it did before.
   2. The water rises less than it did before.
   3. The balloon expands out.
   4. The balloon is sucked in.
7. A student put a drop of blood on a microscope slide and then looked at the blood under a microscope. As you can see in the diagram below, the magnified red blood cells look like little round balls. After adding a few drops of salt water to the drop of blood, the student noticed that the cells appeared to become smaller. This observation raises an interesting question: Why do the red blood cells appear smaller?
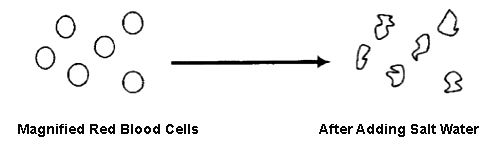


Here are two possible explanations: I. Salt ions (Na^+^ and Cl^-^) push on the cell membranes and make the cells appear smaller. II. Water molecules are attracted to the salt ions so the water molecules move out of the cells and leave the cells smaller.

To test these explanations, the student used some salt water, a very accurate weighting device, and some water-filled plastic bags, and assumed the plastic behaves just like red-blood-cell membranes. The experiment involved carefully weighting a water-filled bag, placing it in a salt solution for ten minutes and then reweighting the bag. *What result for the experiment would best show that explanation I is probably wrong*?

1. the bag loses weight
2. the bag weighs the same
3. the bag appears smaller
4. *What result of the experiment would best show that explanations II is probably wrong*?
   1. The bag loses weight
   2. The bag weighs the same
   3. The bag appears smaller

**Religiosity**

*Religious Practice*

Use the following scale:

1 – More than once a day, 2 – Once a day, 3 – More than once a week, 4 – Once a week, 5 – More than once a month, 6 – Less than once a month

1. How often do you read holy scriptures?
2. How often do you attend Sunday School, religious classes, or seminars?
3. How often do you pray?
4. How often do you attend organized worship services?
5. How often do you attend other activities sponsored by a religious group?

*Religious Influence*

Use the following scale:

1 – No influence at all, 2 – Hardly any influence, 3 – Some influence, 4 – Moderate influence, 5 – A lot of influence, 6 – Extreme influence

1. How much influence do your religious beliefs have on what you wear?
2. How much influence do your religious beliefs have on what you eat and drink?
3. How much influence do your religious beliefs have on your choices about whom you associate with?
4. How much influence do your religious beliefs have on what social activities you undertake?
5. To what extent do your religious beliefs impact the important decisions that you make?

*Religious Hope*

Use the following scale:

1 – Strongly disagree, 2 – Disagree, 3 – Somewhat disagree, 4 – Somewhat agree, 5 – Agree, 6 – Strongly agree

1. There is a heaven.
2. It is possible for all humans to live in harmony together.
3. Miracles are real.
4. My suffering will be rewarded.
5. In the future, my children will be able to lead a better life than me.

**Evolution Acceptance**

Scale: Strongly Disagree, Disagree, Undecided, Agree, Strongly Agree

*Macroevolution*

1. I think that new species *evolved* from ancestral species.
2. I think that the fossil evidence that scientists use to support evolutionary theory is weak and inconclusive.
3. There are a large number of fossils round all around the wolrd that support the idea that organisms *evolve into new species* over time.
4. I think all complex organisms evolved from a single celled organisms.
5. I think that new species evolve from a lot of small changes occurring over relatively long periods of time.
6. There is little or no observable evidence to support the theory that describes how one species of organism evolves from *a different* ancestral form.
7. The forms and diversity of organisms have changed dramatically over time.
8. I think that all organisms are related (or share a common ancestor)

*Microevolution*

1. I think that organisms, as they exist now, are perfectly adapted to their natural environments and so will not continue to change.
2. All groups of organisms will continue to change.
3. There are a large number of examples of organisms that have undergone evolutionary *changes with the species* (i.e., antibiotic resistance in bacteria, production of new strains of the flu virus).
4. Species were created to be perfectly suited to their environment, so they do not change.
5. I don't accept the idea that a species of organism will evolve new traits over time.
6. I think there is an abundance of observable evidence to support the theory describing how *variations within* *a* species can *happen*.
7. *Species* exist today in exactly the same shape and form in which they always have.
8. There is overwhelming evidence supporting the theory of evolution to explain how variations in a species develop over time.

*Human Evolution*

1. There is reliable evidence to support the theory that describes how humans were derived from ancestral primates.
2. Although humans may adapt, humans have not/do not evolve.
3. I think the physical structures of humans are too complex to have evolved.
4. I think that humans and apes share an ancient ancestor.
5. I think that humans evolve.
6. Humans do not evolve; they can only change their behavior.
7. The many characteristics that humans share with other primates (i.e., chimpanzees, gorillas) can best be explained by our sharing a common ancestor.
8. Physical variations in humans (i.e., eye color, skin color) were derived from the same processes that produce variation in other groups of organisms.

**Evolution Knowledge/Misconceptions**

Scale: Strongly Disagree, Disagree, Somewhat Disagree, Neither Agree nor Disagree, Somewhat Agree, Agree, Strongly Agree

*Creationism*

1. I read the bible literally.
2. The Earth isn’t old enough for evolution to have taken place.
3. There was a time when humans and dinosaurs lived on earth together.
4. Present animal diversity can be explained by the Great Flood.
5. Adam and Eve of Genesis are our universal ancestors of the entire human race.
6. All modern species of land vertebrates are descended from those original animals on the ark.

*Conflict Model*

1. People who accept evolution as fact are immoral.
2. If you accept evolution, you really can’t believe in God.
3. Darwinism strips meaning from our lives.

*Knowledge*

1. In most populations, more offspring are born than can survive.
2. Mutations can be passed down to the next generation.
3. Increased genetic variability makes a population more resistant to extinction.
4. The more recently species share a common ancestor, the more closely related they are.
5. Mutations occur all the time.
6. Characteristics acquired during the lifetime of an organism are passed down to that individual’s offspring.
7. Evolution means progression toward perfection.
8. Evolution is a linear progression from primitive to advanced species.

*Revised Knowledge (Classroom Intervention)*

1. According to science, in most populations, more offspring are born than can survive.
2. According to science, mutations can be passed down to the next generation.
3. According to science, increased genetic variability makes a population more resistant to extinction.
4. According to science, the more recently species share a common ancestor, the more closely related they are.
5. According to science, mutations occur all the time.
6. According to science, characteristics acquired during the lifetime of an organism are passed down to that individual’s offspring.
7. According to science, evolution means progression toward perfection.
8. According to science, evolution is a linear progression from primitive to advanced species.
