# Supplementary material for: Conflation between knowledge and acceptance may contribute to the knowledge gap between Judeo-Christian and non-religious people
Source: PLoS One. 2026 Jan 2;21(1):e0319962. doi: 10.1371/journal.pone.0319962 (PMC12758684; doi:10.1371/journal.pone.0319962)
Supplement: Attachment — Submitted filename: 25 05 13 - PLOS one review .docx [file pone.0319962.s003.docx]

The research article by Hodson et al. addresses a critical issue in evolution education by examining the relationship between evolution acceptance and knowledge, particularly among Christian and non-religious students. The authors explore the potential conflation of these two constructs and investigate how perceived conflict with religious beliefs may influence how students respond to evolution knowledge assessments. This review evaluates the study’s significance, introduction, methodology, results, and conclusions.

**Significance of the Study:**

This study makes a novel contribution by shifting focus from simply measuring the relationship between evolution knowledge and acceptance to exploring the possibility that these constructs may be conflated, particularly among religious students. While previous research has often compared differences between religious and non-religious students, this study goes further by experimentally probing the effects of perceived conflict on students’ responses by the inclusion of a quasi-experimental classroom component. This methodological innovation provides valuable insight into how religious identity may influence not only evolution acceptance, but also the expression of evolution knowledge. Despite its strengths on significant of the study, there are several aspects that would benefit from clarification and further development. Therefore, I recommend a **major revision** to strengthen the work.

**Introduction:**

Lines 50–54. The sentence beginning with *“This research has shown…”* would benefit from clarification. The pronoun “this” is ambiguous, does it refer to the authors' current study or to the body of prior literature? I suggest the author to name the research explicitly (e.g., “Prior survey research of general U.S. population (Dunk et al., 2017)…”). Also, the claim that “*Christian religious affiliation is a major predictor of evolution rejection”* should be more carefully framed. While Dunk et al. (2017) did find a statistically significant relationship between religious denomination and evolution acceptance using a general linear model, the effect size reported suggests a small effect. Although the religious denomination contributes to the variance, but it may not be appropriate to characterize it as a “major” predictor without further qualification. The authors could strengthen this section by explicitly reporting the size and context of the effect.

Lines 57–59. The authors summarize that *“affiliation with and commitment to Christian, or biblically grounded, religious belief is likely to hinder the acceptance of evolution.”* This conclusion, while supported by survey data such as the Pew reports, would benefit from contextualization. Since the focus here is on data from the United States, it would be important to reiterate that these trends are culturally and geographically situated, and may not generalize to global patterns of evolution acceptance.

Lines 60–78 (2nd–3rd paragraphs). Before discussing into the complex relationship between religion and evolution, the introduction would benefit from a clear and early articulation of how the authors conceptualize and operationalize “evolution acceptance” and “evolution rejection.” Referring to existing literature that defines these constructs, such as Barnes et al. (2021) that reviews the validity of current evolution acceptance instruments, would add conceptual clarity and strengthen the theoretical framing. Additionally, Lines 63-66, it would be useful if the authors specified which dimensions of religious belief (e.g., belief in God, religious teaching, religious community) are most significant conflict predictor of evolution acceptance.

Lines 79–94 (4th paragraph). Prior work that models’ interactions between knowledge and religiosity is omitted. The author could cite U.S. public-sample research (Weisberg et al., 2018) and student-sample work (Aini et al., 2024) showing that knowledge predicts acceptance more strongly among individuals with lower religiosity. This literature better supports author’s argument that the knowledge–acceptance link is conditionally moderated by religious variables.

Lines 95–111. While this section is a strength especially stating the hypothesis that knowledge scores may be conflated with acceptance is clearly articulated, there are major area of improvement especially in the terms of structure sentences. In some sentences, it blurs the boundary between the *introduction of hypotheses* and *discussion of supporting evidence*. Rather than citing previous studies as *support* for the hypothesis at this stage (e.g., Mead et al., 2018; Winslow et al., 2011), it would be more appropriate to present those findings as part of the rationale for the current research. This would avoid giving the impression that the hypothesis has already been confirmed. Reorganizing this section to clearly separate the hypothesis from the discussion of prior findings would enhance clarity and maintain a logical flow.

Lines 112–115. The sentence describing the first study (*“the first study examined these relationships on a large scale through administration of a survey targeting evolution knowledge, evolution acceptance, scientific reasoning skills, and religiosity”*) feels more appropriate for the *Methods* section. It can be confusing for the readers since it introduces "scientific reasoning skills" as a variable of interest, even though earlier in the introduction the authors seem to downplay or dismiss its relevance to evolution acceptance.

**Methods:**

Lines 135–137. While attention checks are useful for improving data quality, they do not constitute formal validity evidence in a psychometric sense (e.g., content, construct, or criterion validity). It would be more appropriate to revise this sentence.

Line 137. Information about the response rate is missing. Including this would help readers assess the potential for nonresponse bias, which can impact the generalizability and representativeness of the findings.

Table 1 / Line 143. The categorization of participants as *“Theist (Judeo-Christian)”* is unclear and potentially problematic. The term “Christian” was consistently used in the introduction, and it would be more informative to retain consistency and specify denominational affiliations. This distinction is important given that prior research (e.g., Dunk et al., 2017) has shown denominational differences in evolution acceptance. Clarifying this terminology will enhance interpretability and theoretical alignment with the literature discussed earlier.

Lines 162–165. The authors report using a subscale from the Evolution Attitudes and Literacy Survey–Short Form (EALS-SF); however, more detail is needed. It is unclear which subscale was used and how many items were included. Providing example items would allow readers to more effectively evaluate the appropriateness of the measure in relation to the study's objectives.

A conceptual concern with this instrument should also be addressed. While the stated aim of the study is to examine whether evolution knowledge and acceptance are being conflated in student responses, the nature of the EALS-SF itself may contribute to this conflation. Specifically, the use of a Likert-type agreement scale to assess knowledge risks capturing attitudinal agreement rather than cognitive understanding. This blending of epistemic and affective constructs is especially problematic in a study focused on disentangling the two.

The authors should also consider discussing the consequential validity of the EALS-SF, especially the implications of interpreting agreement scores as indicators of a student's knowledge, comprehension, or application of evolutionary theory, rather than as expressions of their acceptance of its scientific validity. This concern is central to the paper’s argument and should be explicitly acknowledged, ideally both in the introduction and in the interpretation of results.

Line 186-190. This sentences that describes the structure and scoring of the evolution knowledge instrument, would be more appropriately placed in the earlier section where the evolution knowledge measure is first introduced since it’s important details from the description of the instrument itself.

Line 194: The authors should specify the estimator used in the confirmatory factor analysis (e.g., maximum likelihood, weighted least squares). The choice of estimator has important implications for model fit and the treatment of ordinal data, especially when Likert-scale responses are used.

**Results**

Lines 218–221: The authors report that participants identifying as *agnostic or atheist* scored significantly higher on the evolution knowledge assessment than participants identifying with *Judeo-Christian religions*. The terminology used across the manuscript lacks consistency. In the introduction and figures, the groups are labeled as “atheist/agnostic” and “religious,” but here the text specifies “Judeo-Christian” in the method or “Christian” in the rest of manuscript. For clarity and alignment across sections, the authors should consistently define and use their grouping terms—e.g., “religious vs. nonreligious,” “Christian vs. atheist/agnostic,” or “Judeo-Christian vs. atheist/agnostic”—and specify how these categories were operationalized. Additionally, consider explaining the rationale for grouping atheists and agnostics together and distinguishing them from religious participants, as these groups may differ in epistemological

views.

Lines 249–253: The authors’ interpretation of predictive power based on standardized factor loadings in the structural equation model is problematic. Factor loadings describe the relationship between latent constructs and their observed indicators and are not valid indicators of predictive strength. Predictive power should be evaluated using standardized path coefficients and the amount of variance explained (e.g., R² values) in the dependent variable. Even if the authors were referring to standardized regression paths, direct comparison of path coefficients across groups requires multi-group SEM with formal testing procedures (e.g., chi-square difference tests with equality constraints). Without this statistical support, the conclusion about differences in predictive power across groups should be interpreted cautiously or revised entirely.

**Discussion:**

Lines 269–271. The discussion begins by stating that *agnostic/atheist respondents agreed more with evolution knowledge items than Christian respondents*. Again, the inconsistent use of group labels (e.g., “Christian,” “Judeo-Christian,” “religious,” “agnostic/atheist,” “nonreligious”) may confuse readers and should be standardized throughout the paper. The authors should clearly define each group and justify the classification scheme.

Lines 279–282: This section of the discussion effectively highlights how inserting the phrase “According to science…” into evolution knowledge items resulted in higher scores, even though the item content remained unchanged. This finding provides compelling evidence for the central claim of the paper, that student responses to knowledge assessments may be influenced by their acceptance of evolution, revealing a possible conflation between conceptual understanding and personal belief. To strengthen this point, the authors should offer a more explicit critique of the instrument used. Specifically, the EALS assesses "evolutionary knowledge" using agreement-based Likert scales, which inherently risk conflating acceptance with understanding. This measurement approach may not fully separate students’ conceptual grasp of evolution from their attitudinal alignment with the statements. To support this critique, the authors could cite studies that have attempted to disentangle knowledge from acceptance using alternative epistemic framings. For instance, the **ACORNS instrument** (Nehm et al., 2012) prompts students to explain how a **biologist** might account for evolutionary phenomena, thereby framing responses explicitly within a scientific context. Research using such instruments has shown that question wording and framing significantly affect how students—particularly those experiencing perceived conflict between science and religion—respond to evolution-related assessments.

Lines 286–316 (Three Explanatory Hypotheses). The authors present three potential explanations for the observed lower knowledge scores among religious students. First, Conflict-induced identity-protective cognition (Kahan et al., 2007), second Stigmatization and disengagement, and last Measurement-induced conflation (students masking understanding due to religious conflict). While the third explanation is central to the study’s contribution, the first two explanations are only loosely connected to the findings and are not empirically supported within this study. These cognitive and affective frameworks would be more appropriately discussed in the introduction, where they could help establish the theoretical foundation for the study’s hypothesis. In contrast, the third explanation—the potential conflation of knowledge and acceptance in survey responses—is directly supported by the study’s findings and should be emphasized more strongly in the discussion.

Lastly, Given the methodological implications of this study, it would be helpful to include a limitations section directly following the Methods. For example, concerns about the psychometric validity of the EALS-SF (e.g., use of Likert scales to assess knowledge), the absence of formal invariance testing in SEM comparisons, and the potential role of social desirability or response bias should be acknowledged upfront. This would allow readers to interpret the results and conclusions within an appropriately cautious framework.
