# Supplementary material for: Conflation between knowledge and acceptance may contribute to the knowledge gap between Judeo-Christian and non-religious people
Source: PLoS One. 2026 Jan 2;21(1):e0319962. doi: 10.1371/journal.pone.0319962 (PMC12758684; doi:10.1371/journal.pone.0319962)
Supplement: Attachment — Submitted filename: Response to Reviewers.docx [file pone.0319962.s005.docx]

| **Reviewer Comment** | **Response to Reviewer** |
| --- | --- |
| **Reviewer #1**: The authors present two studies – a national cross-sectional study and a large experimental study – to examine the relationships between evolutionary acceptance, evolutionary knowledge, and religiosity. Together, these studies are interesting and suggest a novel possibility that Christians suppress knowledge about evolution because it is conflated with acceptance of evolution (and suppression reduces potential conflict between knowledge and acceptance). That being said, I don’t feel this study is currently ready for publication. I’m not sure whether its contribution warrants publication at PlosONE in terms of scope of impact, but I gladly defer to the editorial team on this. I certainly would like to see a more robust, revised version published, and I would be happy to review any revisions. I believe the authors can address my comments with revisions, and I think this distinction could contribute to the broader scholarship in this area. | Thank you. We hope that the revisions have made the paper robust enough to warrant publication |
| 1. The first study is simple, but I am left wondering why some variables were unmeasured, given the literature review. The authors say that “the first study presented in this manuscript aimed to establish clear relationships between evolution knowledge, religiosity, and evolution acceptance.” (lines 98-99) However, the literature review rightly pointed to several nuanced factors that impact this relationship (understanding of the nature of science, political ideology, perceived conflict between science (or evolutionary theory specifically), and religion (or their religious beliefs specifically)). I don’t quite know how to interpret the findings, given that these things were not included. I’m not convinced these relationships were established clearly because there are clear influences that were not accounted for. (Although I can note that some things should have been measured, it’s not clear that political ideology – which was measured – served any purpose beyond demographic information. I’m also unclear why this choice was made.) | Thank you for pointing out this ommision. We actually did gather quite a bit more data and in our efforts to simplify the findings, we inadvertantly omitted important information. We did measure scientific reasoning ability, religiosity, and political ideology. We did not measure perceived conflict as the pCORE (a great instrument for measuring this conflict) was not published when we gathered these data. We did include a few questions from the EALS that sort of gets at conflict but they proved to be rather unreliable and didn't make sense to include in our analysis. We did not measure nature of science understanding, although we wish we had. We have gone back and re-run analyses including the religiosity, ideology, and education level variables and I do believe it significantly adds to the depth of these relationships. We did not, however, include the scientific reasoning test (see next comment). We appreciate the reviewer for pointing this out. |
| 2. Line 115 indicated that scientific reasoning skills were part of the first study. I don’t see this. Scientific reasoning is also mentioned in study 2 in the context of assumptions made about the students' reasoning skills (see line 274). I think this needs to be clarified and addressed. | While we did attempt to measure scientific reasoning ability (just to confirm previous findings of no relationship), we ultimately decided to omit it from analyses due to validity issues. We have briefly stated this in the paper, but we are happy to provide you with additional details here: First, the first four items were non-descriminatory because every respondent got them correct. (This was worrisome in and of itself given that it is unusal for all respondents to get all correct, so we may have had a technical error here). An additional item (item 12) had all respondents getting it wrong. Additionally, the correlational reasoning construct was only measured by two items (this was how the original instrument was designed) and thus could not be included in a CFA if we parsed it out be reasoning abilty as factors. Additionally, two of the four items on the Hypothetico-deductive construct (which, incidentally is an important construct that distinguishes formal and post-formal reasoners from concrete reasoners), showed responses that were no better than guessing and we worried that respondents encountered these longer and more complicated items and they did not try. Lastly, we looked at the average scores on the reasoning test (including all 24 items) by educational level and found that the average ranged from 10 to 14 out of 24. In previous work that we have done (and others; see Jensen, J.L., et al. 2018. Investigating strategies for pre-class content learning in a flipped classroom, Journal of science Education and Technology, 27, 523-535; Lawson, A. E. et al., 2000. What kinds of scientific concepts exist? Concept construction and intellectual development in college biology, Journal of Research in Science Teaching, 37, 996-1018; Jensen, J. L. et al., 2015, Learning scientific reasoning skills may be key to retention in science, technology, engineering, and mathematics, Journal of College Student Retention: Research, Theory, & Practice, 0, 1-19.), we have found these levels (especially for those with advanced degrees) to be far lower than we would expect, again leading us to believe that participants may have just guessed on these more complicated items. |
| 3. The discussion raises several possibilities about what the phrase “according to science” is doing to raise evolutionary knowledge. This, I think, is the most interesting contribution of this study. It’s also an area where I think more discussion is required; I don’t think the authors have the data to support their claim (as currently made) and I think other possibilities are not yet ruled out. I’ll share some thoughts here for the authors’ consideration (and to potentially dialogue with). |  |
| a. The third option, favored by the authors, assumes the lower knowledge scores without the “according to science” are purposeful (line 338). I don’t think the data support this. It could be purposeful, but it could just as easily be unconscious or unintentional. For example, the phrase could activate a different memory schema (e.g., a form of context-dependent memory) that activates the evolutionary knowledge structures in place more strongly. However, for the most religious, this activation may require prompting to override a “default” religious interpretation/knowledge structure. Whether it is intentional or not is a separate empirical question. | This is a fascinating idea and one that we had not considered! We have added to our discussion this possibility along with the possibilitiy suggested in "b". Very inciteful. |
| b. There was no mention of trust in science or trust in scientists (which don’t seem to operate the same). How might trust in either impact (or interact) with reasoning according to science? Political affiliation (and specific denominational affiliations) might matter here quite a bit. For some, asking “according to science” might not mitigate potential conflict (as mentioned in line 336), but might exacerbate it (e.g., equipping an anti-evolution apologetic structure; reasoning is improved, but the conflict is also widened). Like the above comment, this is an empirical question, and the authors don’t have the data to decide between these options. | Thank you for this suggestion. We have expanded our discussion. Unfortunately, we do not have political affilitation for these students (and almost all would have been affiliated with the CJCLDS church, so there are no differences there). But, it is interesting to suggest that it perhaps prompted a negative affect (as suggested in c). We have included this possibility. Brilliant! |
| c. Asking for responses “according to science” could also activate different identity/social systems. Was there an interaction with gender (a sloppy proxy for empathy)? I would also be interested in ruling out the possibility that asking “according to science” did something with social identity, “reasoning as if I were a scientist” rather than demonstrating stable knowledge. For example, if the participants don’t see themselves as especially science-inclined, reasoning according to science could just mean “the opposite of what I think” leading unknowing individuals into a more correct response than otherwise possible. | We unfortunately do not have any identifying information for these students beyond the aggregate student demographics mentioned in the paper. The survey was purposefully anonymous. But, I do like the possibility of "the opposite of what I think" playin a role. We have included that. |
| 4. One limitation discussed was using a Likert scale measure for evolution knowledge. Given this, some intentional attention to the directions would be helpful. Do they understand this as a knowledge test? (The instructions are not included with the question in the instructional materials, but given that the experimental manipulation was with instructions, this is a significant omission.) | Great catch. There was no specific instruction preceding the knowledge questions that would look any different than the acceptance questions. We agree that this is very important to point out. It further strengthens the need to be precise in our instrumentation when we measure "knowledge of evolution". We have added this detail to our methods section (and included the full survey in the supplement). |
| Other comments: |  |
| 1. Were the demographic descriptions of the nationwide sample collected as part of the study, or were these the screener criteria from Qualtrics? This should be clarified (and question wording provided, if relevant). I’m accustomed to more robust participant descriptions and find myself feeling the lack of that in both of these studies. | They were collected as part of the survey (rather than as screening). We have clarified that in the manuscript. And, great catch! We should have included the full survey in the supplement. We have done that now so wording can be assessed. |
| 2. Although the questions to measure religiosity were provided in the supplemental materials, I did not see any statistical validation of these measures in this sample. Since not all of the questions are decidedly religious (e.g., my children will have a better life), this seems important. | We apologize for the oversight. We have amended the supplement to include both the full survey and the full CFA statistics for each measure, including religiosity. |
| 3. Although evolution acceptance was lower among religious respondents in the national sample, it seemed higher than I would have expected, given previous research. Is that true? Contextualizing the size of the effects (within this study and relative to previous studies) would be helpful. (This comment also applies to the ~3% increase in the classroom study when asked to answer “according to science” – where did this move reasoning, relative to the atheist sample in the national survey or previous research?) | Regarding evolution acceptance at the nationwide level, I don't believe the numbers are surprising. Accroding to poll data we cited in the intro for contextualization, nationwide acceptance is around 60% for the general public, and closer to 82% if you include a theistic evolution viewpoint. And amongst Christians, that number ranges between 42 and 64% when just looking at human evolution. We got an average of 96 on a 138-point scale (given the low is 23), which included micro, marco, and human evolution. This would indicate a value very close to what we are seeing nationwide. However, regarding the 3% increase in the classroom study, this is a valid point to consider. It is a small increase, to be sure, but it was also a very small intervention. It is difficult to contextualize this given that other studies use different instruments, so I'm unsure as to how to compare it to others, as well as the fact that we did an entirely new intervention that has not been done. |
| 4. In the national sample, one item for evolutionary acceptance didn’t load for the religious sample, and a different one didn’t load for the atheist/agnostic sample. Why was this? What do you think was happening with this question in these groups? This was never discussed. | This is a good question. Unfortunately, looking at the two items, we can't really come up with an explanation to adequately explain why these would be different between religious and non-religious populations. We suspect it is just due to random messiness in the data, as the fit was very close on each item. We are not sure that a discussion of this would be helpful to the reader. |
| 5. Although the college sample may be homogenous religiously, the national sample combines huge swaths of “Christian tradition” that may not be best understood as a singular group. (Same thing with atheists/agnostics, too.) I think some attention to these potential differences – and some assurance to your reader that it is right to combine them for these analyses – is important. “Christians” can include denominations that explicitly accept (and endorse) evolution and those that are staunchly against it. (This is even true within the same denomination, with more conservative/liberal factions.) Sometimes getting too detailed in these things can make us lose sight of the forest amidst the trees but combining them without asking these questions first can also obscure important differences. | this is an excellent point. We have run analyses for each outcome variable separately between religious affiliations and between agnostic/atheist respondents. There are differences that we have included in a descriptive Table (Table 2). Many of the religious groups do not have a representative sample to draw any definitive conclusions about them. We eliminated all religions that had 10 or fewer respondents (a natural break in the data) and re-ran all analyses to see if the patterns changed significantly. They did not, so we opted to maintain all the data. However, we have made notes on this throughout the results, and included Table 2 so people can really assess the data in context. We appreciate the suggestion. |
| **Reviewer #2**: The research article by Hodson et al. addresses a critical issue in evolution education by examining the relationship between evolution acceptance and knowledge, particularly among Christian and non-religious students. The authors explore the potential conflation of these two constructs and investigate how perceived conflict with religious beliefs may influence how students respond to evolution knowledge assessments. This review evaluates the study’s significance, introduction, methodology, results, and conclusions. |  |
| Significance of the Study: |  |
| This study makes a novel contribution by shifting focus from simply measuring the relationship between evolution knowledge and acceptance to exploring the possibility that these constructs may be conflated, particularly among religious students. While previous research has often compared differences between religious and non-religious students, this study goes further by experimentally probing the effects of perceived conflict on students’ responses by the inclusion of a quasi-experimental classroom component. This methodological innovation provides valuable insight into how religious identity may influence not only evolution acceptance, but also the expression of evolution knowledge. Despite its strengths on significant of the study, there are several aspects that would benefit from clarification and further development. Therefore, I recommend a major revision to strengthen the work. | Thank you, we hope our revisions provided the clarification you were hoping to see. |
| Introduction: |  |
| Lines 50–54. The sentence beginning with “This research has shown…” would benefit from clarification. The pronoun “this” is ambiguous, does it refer to the authors' current study or to the body of prior literature? I suggest the author to name the research explicitly (e.g., “Prior survey research of general U.S. population (Dunk et al., 2017)…”). Also, the claim that “Christian religious affiliation is a major predictor of evolution rejection” should be more carefully framed. While Dunk et al. (2017) did find a statistically significant relationship between religious denomination and evolution acceptance using a general linear model, the effect size reported suggests a small effect. Although the religious denomination contributes to the variance, but it may not be appropriate to characterize it as a “major” predictor without further qualification. The authors could strengthen this section by explicitly reporting the size and context of the effect. | The language around the effect of religious affiliation has been made less extreme, removing "major". Also, the pronoun use here was clarified to not refer to the present study, replacing "This" with "Prior" research. |
| Lines 57–59. The authors summarize that “affiliation with and commitment to Christian, or biblically grounded, religious belief is likely to hinder the acceptance of evolution.” This conclusion, while supported by survey data such as the Pew reports, would benefit from contextualization. Since the focus here is on data from the United States, it would be important to reiterate that these trends are culturally and geographically situated, and may not generalize to global patterns of evolution acceptance. | The statement was modified to refer specifically to the US context in order to not extrapolate to a global claim. |
| Lines 60–78 (2nd–3rd paragraphs). Before discussing into the complex relationship between religion and evolution, the introduction would benefit from a clear and early articulation of how the authors conceptualize and operationalize “evolution acceptance” and “evolution rejection.” Referring to existing literature that defines these constructs, such as Barnes et al. (2021) that reviews the validity of current evolution acceptance instruments, would add conceptual clarity and strengthen the theoretical framing. Additionally, Lines 63-66, it would be useful if the authors specified which dimensions of religious belief (e.g., belief in God, religious teaching, religious community) are most significant conflict predictor of evolution acceptance. | At the end of paragraph 1, we added more concrete definitions of evolution acceptance and rejection, specifically pointing out the sometimes partial acceptance, to the exclusion of human evolution. Additionally, in paragraph 2 we added more specificity with what has been shown to predict evolution rejection among religious individuals when other more broad predictors are controlled for. |
| Lines 79–94 (4th paragraph). Prior work that models’ interactions between knowledge and religiosity is omitted. The author could cite U.S. public-sample research (Weisberg et al., 2018) and student-sample work (Aini et al., 2024) showing that knowledge predicts acceptance more strongly among individuals with lower religiosity. This literature better supports author’s argument that the knowledge–acceptance link is conditionally moderated by religious variables. | Additional studies were added to paragraph 4 illustrating the complex nature of connecting knowledge and acceptance based on religious affiliation/religiosity, in order to more clearly illustrate the need to examine mediating factors. |
| Lines 95–111. While this section is a strength especially stating the hypothesis that knowledge scores may be conflated with acceptance is clearly articulated, there are major area of improvement especially in the terms of structure sentences. In some sentences, it blurs the boundary between the introduction of hypotheses and discussion of supporting evidence. Rather than citing previous studies as support for the hypothesis at this stage (e.g., Mead et al., 2018; Winslow et al., 2011), it would be more appropriate to present those findings as part of the rationale for the current research. This would avoid giving the impression that the hypothesis has already been confirmed. Reorganizing this section to clearly separate the hypothesis from the discussion of prior findings would enhance clarity and maintain a logical flow. | As suggested, research framing the hypothesis (the Mead and Winslow studies) was moved up to the prior paragraph to eliminate this confusion. |
| Lines 112–115. The sentence describing the first study (“the first study examined these relationships on a large scale through administration of a survey targeting evolution knowledge, evolution acceptance, scientific reasoning skills, and religiosity”) feels more appropriate for the Methods section. It can be confusing for the readers since it introduces "scientific reasoning skills" as a variable of interest, even though earlier in the introduction the authors seem to downplay or dismiss its relevance to evolution acceptance. | The study framing and survey descriptions has been moved down to methods, in the respective sections for each study. |
| Methods: |  |
| Lines 135–137. While attention checks are useful for improving data quality, they do not constitute formal validity evidence in a psychometric sense (e.g., content, construct, or criterion validity). It would be more appropriate to revise this sentence. | The statement claiming validity through attention checks was removed, instead just stating that it helped ensure their answers weren't random. |
| Line 137. Information about the response rate is missing. Including this would help readers assess the potential for nonresponse bias, which can impact the generalizability and representativeness of the findings. | Unfortunately, this survey was administered through the Qualtrics survey platform. We do not get any data on response rate. They send out random samples to populations until the quota is reached. Thus, we do not have this information. |
| Table 1 / Line 143. The categorization of participants as “Theist (Judeo-Christian)” is unclear and potentially problematic. The term “Christian” was consistently used in the introduction, and it would be more informative to retain consistency and specify denominational affiliations. This distinction is important given that prior research (e.g., Dunk et al., 2017) has shown denominational differences in evolution acceptance. Clarifying this terminology will enhance interpretability and theoretical alignment with the literature discussed earlier. | We have maintained "Judeo-Christian" because our sample included both Christian and Jewish respondents. However, we definitely agree that denominational differences are present. Based on the first reviewer's suggestions, we have expanded our analyses to look at differences in denominations. Unfortunately, many of the denominations do not have a large respresentative sample making it impossible to do denominationally-specific analyses. However, we did eliminate those with fewer than 10 representatives and re-run analyses. Relationships were maintained, so we maintained our full Judeo-Christian dataset. We have, however, included tables with the descriptive information so the reader is more informed about denominational differences present in the data. Thank you for pointing this out. |
| Lines 162–165. The authors report using a subscale from the Evolution Attitudes and Literacy Survey–Short Form (EALS-SF); however, more detail is needed. It is unclear which subscale was used and how many items were included. Providing example items would allow readers to more effectively evaluate the appropriateness of the measure in relation to the study's objectives. | We did list the subscale: it is called the "Evolutionary Knowledge" subscale. But we have also now included the full survey in the supplement. We apologize for the omission. |
| A conceptual concern with this instrument should also be addressed. While the stated aim of the study is to examine whether evolution knowledge and acceptance are being conflated in student responses, the nature of the EALS-SF itself may contribute to this conflation. Specifically, the use of a Likert-type agreement scale to assess knowledge risks capturing attitudinal agreement rather than cognitive understanding. This blending of epistemic and affective constructs is especially problematic in a study focused on disentangling the two. | We agree. This is one of the points we are trying to make. The EALS is used as a knowledge test, but it is worded in a way that can easily conflate it with acceptance. We have also discussed this extensively as a limitation. |
| The authors should also consider discussing the consequential validity of the EALS-SF, especially the implications of interpreting agreement scores as indicators of a student's knowledge, comprehension, or application of evolutionary theory, rather than as expressions of their acceptance of its scientific validity. This concern is central to the paper’s argument and should be explicitly acknowledged, ideally both in the introduction and in the interpretation of results. | Thank you for this suggestion. We have added some discussion about this instrument to our discussion section. |
| Line 186-190. This sentences that describes the structure and scoring of the evolution knowledge instrument, would be more appropriately placed in the earlier section where the evolution knowledge measure is first introduced since it’s important details from the description of the instrument itself. | We were a little confused by this. The scoring for each instrument is in its appropriate section. Perhaps you just misread it. |
| Line 194: The authors should specify the estimator used in the confirmatory factor analysis (e.g., maximum likelihood, weighted least squares). The choice of estimator has important implications for model fit and the treatment of ordinal data, especially when Likert-scale responses are used. | We have included this in our methods section. Thank you. |
| Results |  |
| Lines 218–221: The authors report that participants identifying as agnostic or atheist scored significantly higher on the evolution knowledge assessment than participants identifying with Judeo-Christian religions. The terminology used across the manuscript lacks consistency. In the introduction and figures, the groups are labeled as “atheist/agnostic” and “religious,” but here the text specifies “Judeo-Christian” in the method or “Christian” in the rest of manuscript. For clarity and alignment across sections, the authors should consistently define and use their grouping terms—e.g., “religious vs. nonreligious,” “Christian vs. atheist/agnostic,” or “Judeo-Christian vs. atheist/agnostic”—and specify how these categories were operationalized. Additionally, consider explaining the rationale for grouping atheists and agnostics together and distinguishing them from religious participants, as these groups may differ in epistemological views. | Thank you for pointing this out. We have gone through and been more consistent in our labels. Additionally, hopefully with the added information about religious denominations included (see Table 2), these other concerns are ameliorated. |
| Lines 249–253: The authors’ interpretation of predictive power based on standardized factor loadings in the structural equation model is problematic. Factor loadings describe the relationship between latent constructs and their observed indicators and are not valid indicators of predictive strength. Predictive power should be evaluated using standardized path coefficients and the amount of variance explained (e.g., R² values) in the dependent variable. Even if the authors were referring to standardized regression paths, direct comparison of path coefficients across groups requires multi-group SEM with formal testing procedures (e.g., chi-square difference tests with equality constraints). Without this statistical support, the conclusion about differences in predictive power across groups should be interpreted cautiously or revised entirely. | We have removed all references to comparisons of predictive strength across groups, given that we established a lack of invariance between populations. This was an overlooked error. Thank you for catching it. |
| Discussion: |  |
| Lines 269–271. The discussion begins by stating that agnostic/atheist respondents agreed more with evolution knowledge items than Christian respondents. Again, the inconsistent use of group labels (e.g., “Christian,” “Judeo-Christian,” “religious,” “agnostic/atheist,” “nonreligious”) may confuse readers and should be standardized throughout the paper. The authors should clearly define each group and justify the classification scheme. | This has been fixed. |
| Lines 279–282: This section of the discussion effectively highlights how inserting the phrase “According to science…” into evolution knowledge items resulted in higher scores, even though the item content remained unchanged. This finding provides compelling evidence for the central claim of the paper, that student responses to knowledge assessments may be influenced by their acceptance of evolution, revealing a possible conflation between conceptual understanding and personal belief. To strengthen this point, the authors should offer a more explicit critique of the instrument used. Specifically, the EALS assesses "evolutionary knowledge" using agreement-based Likert scales, which inherently risk conflating acceptance with understanding. This measurement approach may not fully separate students’ conceptual grasp of evolution from their attitudinal alignment with the statements. To support this critique, the authors could cite studies that have attempted to disentangle knowledge from acceptance using alternative epistemic framings. For instance, the ACORNS instrument (Nehm et al., 2012) prompts students to explain how a biologist might account for evolutionary phenomena, thereby framing responses explicitly within a scientific context. Research using such instruments has shown that question wording and framing significantly affect how students—particularly those experiencing perceived conflict between science and religion—respond to evolution-related assessments. | The limitations section has been expanded to recommend contextualizing questions within a given sphere, such as the Nehm study asking participants to answer like a scientist would. |
| Lines 286–316 (Three Explanatory Hypotheses). The authors present three potential explanations for the observed lower knowledge scores among religious students. First, Conflict-induced identity-protective cognition (Kahan et al., 2007), second Stigmatization and disengagement, and last Measurement-induced conflation (students masking understanding due to religious conflict). While the third explanation is central to the study’s contribution, the first two explanations are only loosely connected to the findings and are not empirically supported within this study. These cognitive and affective frameworks would be more appropriately discussed in the introduction, where they could help establish the theoretical foundation for the study’s hypothesis. In contrast, the third explanation—the potential conflation of knowledge and acceptance in survey responses—is directly supported by the study’s findings and should be emphasized more strongly in the discussion. | Per this suggestion, we introduce the identity-protective cognition and stigmatization theories in the introduction of the manuscript as pieces of the hypothesis moving into the study. |
| Lastly, Given the methodological implications of this study, it would be helpful to include a limitations section directly following the Methods. For example, concerns about the psychometric validity of the EALS-SF (e.g., use of Likert scales to assess knowledge), the absence of formal invariance testing in SEM comparisons, and the potential role of social desirability or response bias should be acknowledged upfront. This would allow readers to interpret the results and conclusions within an appropriately cautious framework. | We have included some of the limitations of instrumentation throughout the methods section (instead of as one limitations section) - we felt that was easier for the reader. We did do invariance testing. It is described int he results section. It has been our experience that this is the section where this is traditionally reported. |
|  |  |
| **From the Editor:** |  |
| 1. Please ensure that your manuscript meets PLOS ONE's style requirements, including those for file naming. The PLOS ONE style templates can be found at | We apologize for formatting errors. We have fixed this. |
| [https://journals.plos.org/plosone/s/file?id=wjVg/PLOSOne_formatting_sample_main_body.pdf and](https://journals.plos.org/plosone/s/file?id=wjVg/PLOSOne_formatting_sample_main_body.pdf) |  |
| <https://journals.plos.org/plosone/s/file?id=ba62/PLOSOne_formatting_sample_title_authors_affiliations.pdf> |  |
| 2. We note that the grant information you provided in the ‘Funding Information’ and ‘Financial Disclosure’ sections do not match. | We have fixed this. |
| When you resubmit, please ensure that you provide the correct grant numbers for the awards you received for your study in the ‘Funding Information’ section |  |
| 3. Thank you for stating the following financial disclosure: | We have included this in our cover letter. |
| [Internal University funding]. |  |
| Please state what role the funders took in the study.  If the funders had no role, please state: "The funders had no role in study design, data collection and analysis, decision to publish, or preparation of the manuscript." |  |
| If this statement is not correct you must amend it as needed. |  |
| Please include this amended Role of Funder statement in your cover letter; we will change the online submission form on your behalf. |  |
| 4. When completing the data availability statement of the submission form, you indicated that you will make your data available on acceptance. We strongly recommend all authors decide on a data sharing plan before acceptance, as the process can be lengthy and hold up publication timelines. Please note that, though access restrictions are acceptable now, your entire data will need to be made freely accessible if your manuscript is accepted for publication. This policy applies to all data except where public deposition would breach compliance with the protocol approved by your research ethics board. If you are unable to adhere to our open data policy, please kindly revise your statement to explain your reasoning and we will seek the editor's input on an exemption. Please be assured that, once you have provided your new statement, the assessment of your exemption will not hold up the peer review process. | We have deposited all data in a public repository and included the accession number. |
