# Supplementary material for: Conflation between knowledge and acceptance may contribute to the knowledge gap between Judeo-Christian and non-religious people
Source: PLoS One. 2026 Jan 2;21(1):e0319962. doi: 10.1371/journal.pone.0319962 (PMC12758684; doi:10.1371/journal.pone.0319962)
Supplement: Attachment — Submitted filename: Response to Reviewers_Final.docx [file pone.0319962.s006.docx]

Reviewer #1: I am grateful to review this revised manuscript. It clarifies many ambiguities and presents a more streamlined presentation. There are, however, a few questions I'll raise, in hopes of encouraging even more clarity. I consider all but the first comment minor.

1. Paper organization. As with the first, both the national sample and the classroom sample are presented as one study. I think this does more harm than good for clarity of communication. I would strongly encourage a Study 1/Study 2 presentation, each with their own (brief) literature review, hypotheses/building rationale, method, analysis, etc. In some cases, a simple, "The same measures were used in the classroom study as in the national study" would suffice. This change will reduce the cognitive load required of the reader to hold all the various pieces in mind, and it will help clarify the value of each part of the paper. For example, in the results, all of the SEM models - were they also run for the classroom study? Why not? If not, why bother give all the students all those measures? Having them separated will allow the authors to more carefully communicate what each study does; what questions they were designed to answer and what they add. Some of the things can be moved to supplemental materials (e.g., the same SEM analyses confirmed similar patterns, see supplemental for full statistical read outs), while leaving the important pieces in the paper narrative. This comment is less about changing content, and more about increasing the ease of engaging that content.

We really loved this suggestion and we have split the manuscript into two “studies” for ease of reading. Thank you for suggesting this.

2. Nature of Science. This is still mentioned in the abstract (first sentence!) and in the literature review. Since it wasn't measured, it seems odd to draw attention to it in these places. It feels like a self-inflicted loose end! Help me, as your reader, situate the research on NoS within your study, even if it isn't measured. Could identity (which you discuss) be a gateway to NoS understanding? (I'm thinking of the research on the #ilooklikeanengineer campaign, though I think other work likely applies.)

Excellent catch. This was simply an error in editing on our part. In a larger study, we did include measures of scientific reasoning, conflict, and NOS, but those were not included in this study. It should have been removed from the abstract. We did highlight the past findings on the relationship between NOS and evolution acceptance by way of background, but we have reduced the emphasis on this factor given, as you appropriately pointed out, that we did not measure this.

3. Table 1. Are the demographics different by religious affiliation? This shows up later in the manuscript (lines 320-326), but it seems they should be reported here for clarity and consistency.

Agreed. This is a great suggestion. We have now presented the demographic table divided by religious affiliation.

4. Survey measures. Were the surveys given in a set order (both studies) or were they randomized? Can priming effects be ruled out?

The surveys (in each study) were all administered in the same order: Nationwide – demographics, scientific reasoning, religiosity, I-SEA, EALS-SF; Classroom – the I-SEA and the EALS-SF were given in two separate surveys at two different times. I’m not sure what priming effects there would be and could only speculate. However, we have included this ordering for the nationwide sample, just so the reader is aware and can hypothesize about potential effects.

5. Figure 3 (and associated analysis). Is there a relationship between religious influence and knowledge of evolution? That doesn't seem to be accounted for in the model.

There was no significant relationship between religious influence and knowledge (at least among Theist respondents – we did not measure religious influence among atheist/agnostic respondents), so we did not include this relationship. We included a short statement on this lack of relationship in the results.

6. Speculation. This wouldn't need to be addressed, though I'll share this comment because I think a marker of good research is work that invites new questions: Related to #5, what do you think the relationship between religious influence and knowledge of evolution ought to be (longitudinally)? The answer to this question might actually help sort out some of the possibilities posed in your paper. If it is primarily social identity at play (vs. cognitive dissonance or "science as encouraging deconversion"), then would we expect religious influence --> knowledge (vs. knowledge --> religious influence)? These aren't necessarily mutually exclusive influences, but I wonder if more attention could be paid to contextualizing these findings in light of bigger questions around religion, sociocultural context, belief, and knowledge. As I said, this could be addressed, though I wouldn't expect it to be given the particular focus/restrictions, etc.

It is an interesting question. However, given that we did not see a relationship between religious influence and knowledge, this particular measure is not super informative. However, I think what you are hinting at, and what we seem to be seeing in our paper (that identity as a religious person is driving the lower knowledge) is a fair point. Unfortunately, I don’t think that our particular measure of “Religious Influence” is appropriate to measure this. Our instrument does measure how much religion influences daily life, but in terms of dress and eating habits, rather than decision making. I would guess that, had we thought of it or had they been available at the time, a better measure would have been the pCORE (Barnes et al., 2021) or the pFEAR (Ferguson et al., 2024) as these get more at the perceived conflict, or at the influence of one’s religion in decision making on controversial topics.

Reviewer #2: I still to believe that this study has the potential to make a novel contribution, particularly by providing evidence on how the relationship between knowledge/understanding and acceptance can be conflated. It also helps shift the conversation from a deficit model toward exploring the psychological processes that shape how knowledge is expressed by religious students.
Despite the strengths of the study and my appreciation for the revisions made, I believe further clarity is needed in the methodology. I remain especially concerned about how the measurement was treated and how the scores were interpreted. I am not yet convinced that the current analytical approach supports the claims being made.

Sampling procedure clarity.
More detail is needed about the sampling procedures for the nationwide study. The manuscript notes the use of the Qualtrics surveying platform, but it is unclear whether Qualtrics was only used to host and distribute the survey or whether only recruitment occurred through Qualtrics. How were participants recruited nationwide through Qualtrics? Without this information, it is difficult to assess the representativeness of the sample, as claimed by the author.

Thank you for pointing this omission out. We have further clarified the sampling procedure and the filters used to get a representative sample.

Instrument clarity.
There is still inconsistency in the description of the instrument. Figures 2 and 3 suggest that four EALS items were used, yet the supplemental materials list eight items (including items from the “misconceptions about evolution” subscale), and line 197 refers to five items. This inconsistency makes it unclear what was actually included in the analyses.

Great catch! During the first revision, based on other comments, we did end up removing the first item from the EALS for poor fit. We failed to update our methods section and our supplement. It has been updated now to indicate that we used only four items. We have left it in the Supplemental File of the original survey, since the original survey included all five *and* included the “misconceptions about evolution” that were not used in this study.

Level of measurement and score interpretation
I appreciate the authors’ revisions acknowledging that the EALS-SF subscale is opinion-based and therefore at risk of conflation with belief. However, several important concerns remain unresolved, particularly regarding score interpretation and its statistical and conceptual implications.
The paper’s central claim is that measured “evolution knowledge” differs across religious groups and how it predicts acceptance. The instrument, however, is based on Likert-type agreement items that were summed into total scores (as described in the methods and statistical results) but sometimes expressed as percentages (as shown in Figure 1 and in the discussion). While using summed Likert scores in t-tests is common practice in our field, especially with multiple items and large samples, it still assumes interval-level properties that are not strictly supported by ordinal data. My greater concern arises from converting these scores into percentages, which suggests ratio-level meaning that the instrument cannot support. For example, the authors reported a “57% knowledge score” in the discussion, which implies a proportion of correct knowledge, when in reality the value represents aggregated agreement ratings. This creates a risk of over-interpreting what the measure actually captures and may mislead readers into equating endorsement with factual knowledge.
To give a concrete example, the finding that adding the phrase “according to science” revealed a divergence between the summed scores for “knowledge” and acceptance is one of the most interesting results of the study. The interpretation, that religious students may filter their responses through a schema tied to religious identity, either deliberately or unconsciously, is important. However, the way the scores are reported risks overstating what the instrument actually captures. For instance, describing a student as having “26% on acceptance but 83% on knowledge” implies a ratio-level interpretation that the measure cannot support. These percentages do not represent proportions of correct knowledge; again they are re-scaled aggregates of ordinal agreement ratings. The value of the classroom study lies in showing that the relationship between acceptance and knowledge scores changes under different framings, but the exact percentages (e.g., 26% vs. 83%) are less meaningful, because they suggest a level of precision and factual correctness that the instrument does not provide. Reporting summed or average scores, providing the statistical relationship, and explicitly cautioning readers about what these values represent would strengthen the evidentiary basis for this interpretation.
In other words, while the qualitative pattern observed (greater divergence under the “according to science” framing) is meaningful, the magnitude and precision implied by percentage scores and statistical tests need further consideration, especially since the instrument was not designed with right/wrong answers or validated for interval-level analysis.

We agree that this is a tricky instrument. We had opted for percentages to make it easier for the reader to interpret. However, we re-ran statistics using summed scores and, as expected, the statistics are exactly the same. So, I don’t think percentages are overstating the difference. However, we agree with you that percentages are probably not appropriate for Likert-style data. It’s always tricky to convey Likert scores well and make them easily interpretable given the ambiguous distance between ratings. So, we thought that perhaps including a stacked bar of the Likert distributions would at least allow the reader to interpret the raw summed scores a little better (see Figure 4).

We also agree that the framing was a little funky, as well. We have gone through and reframed the results to reflect a better representation of Likert-style data.

Interpretive framing
I wonder if using average Likert scores or consistently using summed scores rather than percentages would change the results. If so, this would suggest that using percentages is indeed overstating the findings. The authors might also reconsider whether the construct is better framed as “evolution understanding” or “perceived understanding of evolution,” rather than “knowledge of evolution.” This would make clearer that the instrument captures willingness to endorse science-framed statements, not factual correctness. Such a reframing would also align more closely with the authors’ psychological interpretation that students may possess knowledge but choose whether or not to reveal it depending on context.

We agree with this and have wrestled with this throughout the course of this study. We have decided to take the recommendation to reframe it as “evolution knowledge agreement” (we played with “perceived knowledge” but perception didn’t seem quite right. It is more of an agreement with scientific statements) to better represent what the EALS is measuring.

Alternative analyses
To further strengthen the analysis, I suggest presenting the distribution of responses across Likert categories for the control versus “according to science” groups, rather than reducing them to percentages. This would more directly illustrate how framing shifts patterns of agreement. The authors could also consider statistical models that respect the ordinal nature of the data (e.g., cumulative logit models), which avoid over-interpreting the numeric distances between Likert points. Finally, scatterplots or density plots could be used to visualize how acceptance and understanding scores relate to one another in the two conditions. This would highlight the divergence without relying on potentially misleading percentage-based claims.

We considered each of these (thank you for the thorough suggestions) and decided that the stacked-bar charts best illustrated the Likert-style data in a way that was easy to interpret for the reader. We could run individual logit models on each Likert item (or goodness of fit tests for distributions), but analyzing the cumulative score seemed more appropriate.
