# Supplementary material for: Conflation between knowledge and acceptance may contribute to the knowledge gap between Judeo-Christian and non-religious people
Source: PLoS One. 2026 Jan 2;21(1):e0319962. doi: 10.1371/journal.pone.0319962 (PMC12758684; doi:10.1371/journal.pone.0319962)
Supplement: Attachment — Submitted filename: Response to Review.docx [file pone.0319962.s007.docx]

Reviewer #1: Thank you for this revision. I appreciate the way you addressed my comments, and those of the other reviewer! I think this is an important paper, well presented, ready for publication.

RESPONSE: Thank you!

Reviewer #2: Thank you for the revised manuscript! I believe this work will make a meaningful contribution to the field. The re-organization of Studies 1 and 2, along with the addition of the new figure, substantially improves clarity and strengthens the overall narrative. I have only a few minor comments that I think will further enhance readability and consistency.

1. Line 121 (clarity): The sentence “In other words, two respondents with an equal knowledge of evolution may score differently on a knowledge instrument depending on their existing level of evolution acceptance” seems intended to clarify the point, but I found it difficult to understand on its own without having read the full paper, especially the Discussion

RESPONSE: Agreed. We thought it best to just delete the sentence. It does not add to the clarity. The point is stated in the sentence before.

2. Table 1 and Line 292 (political ideology coding consistency): In Table 1, political ideology is presented in three categories (conservative-leaning, moderate, liberal-leaning), but the text around Line 292 appears to reference analyses based on the original 7-point scale (also described earlier as a 7-point Likert measure). I understand why you collapsed political ideology into three groups for Table 1, but I suggest clarifying this more explicitly and ensuring consistent wording across the manuscript. For example, you could state that Table 1 uses collapsed categories for descriptive purposes, while analyses use the full 7-point measure (if that is the case).

RESPONSE: Good catch! I’m not sure why we collapsed it in the table; I suppose just for ease of interpretation. I’ve gone ahead and expanded it in the table to match all our analyses and avoid any confusion.

3. Include student instructions in the supplement (replicability and instructional value).
I recommend adding the exact instructions/prompts used in the study to the supplemental materials. Because the manuscript highlights the impact of adding the phrase “according to science,” including the full wording would improve transparency and make it easier for instructors and researchers to replicate or adapt the approach in future work.

RESPONSE: We have included the modified survey in the supplemental file 1. That is a good idea. There were no additional instructions or prompts. The survey was simply administered just as it appears in the supplement. So, we have nothing else to include.

4. Line 515 (add nuance about the sample/group).
The sentence would benefit from a small clarification to reflect the specific context in which this result was observed. I suggest adding wording that indicates this improvement was found in a particular study/sample (or subgroup) so the claim does not read as universally generalizable across all groups.

RESPONSE: Thank you. We added further clarification: “…as was done in this study using the EALS instrument with undergraduate Christian students…”
